# Hyperpolarized ^13^C MRI uncovers early and progressive metabolic dysfunction in the hAPP-J20 mouse model of Alzheimer’s disease

**DOI:** 10.21203/rs.3.rs-10437814/v1

**Published:** 2026-07-27

**Authors:** Tamara Vasilkovska, Lydia M. Le Page, Caroline Guglielmetti, Marina Radoul, Huihui Li, Xiao Ji, Jeremy W. Gordon, Ken Nakamura, Myriam M. Chaumeil

**Affiliations:** University of California, San Francisco; University of California, San Francisco; University of California, San Francisco; University of California, San Francisco; Gladstone Institutes; University of California, San Francisco; University of California, San Francisco; Gladstone Institutes; University of California, San Francisco

**Keywords:** hyperpolarized 13C MRSI, Alzheimer’s disease, brain metabolism, amyloid beta, biomarker, metabolomics

## Abstract

Impaired brain energy metabolism is an early feature of Alzheimer's disease (AD), but the standard metabolic imaging tool, [^18^F]FDG-PET, reports only glucose uptake and cannot resolve downstream metabolic flux nor inform on overall metabolic profile of the brain. In this study, we used [^18^F]FDG-PET, hyperpolarized (HP) [1-^13^C]pyruvate ^13^C magnetic resonance spectroscopic imaging (MRSI), and ex vivo ^1^H-NMR metabolomics to characterize how amyloid-β (Aβ) associated pathology reshapes brain energy metabolism in the hAPP-J20 mouse model, from glucose uptake to metabolic fluxes and steady-state metabolite concentrations. HP ^13^C MRSI was performed in male and female wild-type (WT) and hAPP-J20 mice at 2 and 14 months of age, with FDG-PET and metabolomics acquired at the final timepoint.

HP ^13^C MRSI revealed a progressive, region-specific increase in glycolytic flux in hAPP-J20 mice, with increased apparent HP [1-^13^C]Lactate/Pyruvate conversion (relative to vascular delivery) localized to the hippocampus in females and to the cortex in males; HP ^13^C urea perfusion measures confirmed comparable substrate delivery between genotypes. In contrast, [^18^F]FDG-PET showed no genotype difference in regional glucose uptake, although the sex- and weight-dependent scaling of FDG uptake observed in the WT controls was lost in hAPP-J20 mice. *Ex vivo* metabolomics uncovered sex-divergent metabolic rewiring: a succinate-centered strengthening of the TCA cycle and propanoate metabolism in females, versus altered tyrosine and ubiquinone metabolism in males.

These data show that HP ^13^C MRSI detects an Aβ-associated increase in glycolytic flux before FDG-PET registers a change, and position HP ^13^C pyruvate MRS imaging as a sensitive, radiation-free, flux-based biomarker that complements glucose-uptake imaging in AD.

## Introduction

Alzheimer’s disease (AD) is characterized by progressive cognitive decline accompanied by widespread synaptic dysfunction and neurodegeneration(1). Alterations in brain energy metabolism represent one of the earliest detectable features of the disease, emerging years before the onset of overt neuronal loss or clinical symptoms(2-5). Reductions in cerebral glucose utilization have been consistently observed in individuals at risk for AD as well as in patients with established disease, particularly within association cortices and hippocampal networks that support memory and cognition (2, 4, 6-9). These metabolic deficits precede measurable neurodegeneration and are thought to contribute to neuronal vulnerability and synaptic dysfunction(10, 11). Consequently, metabolic alterations are increasingly recognized as central components of AD pathophysiology rather than merely secondary consequences of neuronal loss.

Non-invasive neuroimaging has played a key role in revealing neuronal and functional alterations associated with AD. In particular, Positron Emission Tomography using fluorodeoxyglucose (FDG-PET) has demonstrated robust reductions in regional glucose uptake in both patients and animal models of AD(2, 4, 9). FDG-PET therefore serves as a widely used biomarker of neuronal metabolic activity, although it does not inform on the steps in glucose metabolism that lead to energy production that are needed to support neuronal activity(12). However, FDG-PET primarily reflects glucose transport and phosphorylation and does not provide direct information about downstream metabolic pathways such as glycolysis, lactate production, or mitochondrial oxidative metabolism(13, 14). As a result, important aspects of metabolic dysfunction—including alterations in metabolic flux and substrate utilization—remain difficult to capture using this approach alone.

Hyperpolarized ^13^C magnetic resonance spectroscopic imaging (HP ^13^C MRSI) is an emerging metabolic imaging technique that enables real-time visualization of enzymatic metabolic fluxes in vivo. By dramatically enhancing the signal of ^13^C-labeled substrates such as [1-^13^C]pyruvate, hyperpolarization allows rapid detection of downstream metabolic products including lactate and bicarbonate, which reflect glycolytic activity and mitochondrial oxidative metabolism(15-17). HP ^13^C MRI provides a unique ability to assess metabolic pathway activity in real time and has shown strong potential for investigating metabolic dysfunction across multiple diseases including neurological disorders(18-21). Recent preclinical studies suggest that hyperpolarized metabolic imaging may reveal alterations in oxidative metabolism and glycolytic flux in models of neurological disorders(19, 22, 23), highlighting its promise as a non-invasive tool for studying brain metabolism in AD.

Animal models of AD provide an important platform for investigating these metabolic processes under controlled conditions. The hAPP-J20 mouse model, which overexpresses human amyloid precursor protein (hAPP) containing familial AD mutations (Swedish and Indiana), exhibits progressive amyloid-β (Aβ) accumulation, synaptic dysfunction, and cognitive deficits that recapitulate key features of AD pathology(24-26). In addition to Aβ pathology, this model displays abnormalities in neuronal network activity and metabolic signaling, including hippocampal hyperexcitability and disrupted neuronal circuit function(27). These features make the J20 model well suited for investigating how Aβ pathology affects brain metabolic regulation.

Despite extensive evidence linking AD to metabolic dysfunction, relatively little is known about how distinct aspects of brain metabolism—glucose uptake, metabolic flux, and metabolite pool composition —contribute to neurodegeneration. A multimodal approach integrating complementary metabolic measurements may therefore provide a more comprehensive understanding of metabolic alterations in AD, while also increasing the sensitivity to detect disease onset and subtypes, and to monitor disease progression and therapeutic response.

In the present study, we combine HP ^13^C MRSI, FDG-PET imaging, and *ex vivo* NMR metabolomics to investigate metabolic alterations in the hAPP-J20 mouse model of Aβ pathology. This integrated approach enables simultaneous assessment of glucose uptake, real-time metabolic flux through glycolytic and oxidative pathways, and steady-state metabolite concentrations within the brain. By leveraging the complementary strengths of these methods, we aim to define how Aβ pathology reshapes brain energy metabolism across multiple levels of organization and to identify metabolic signatures that may contribute to neuronal dysfunction in AD.

## Methods

### Animals

**Figure 1:**
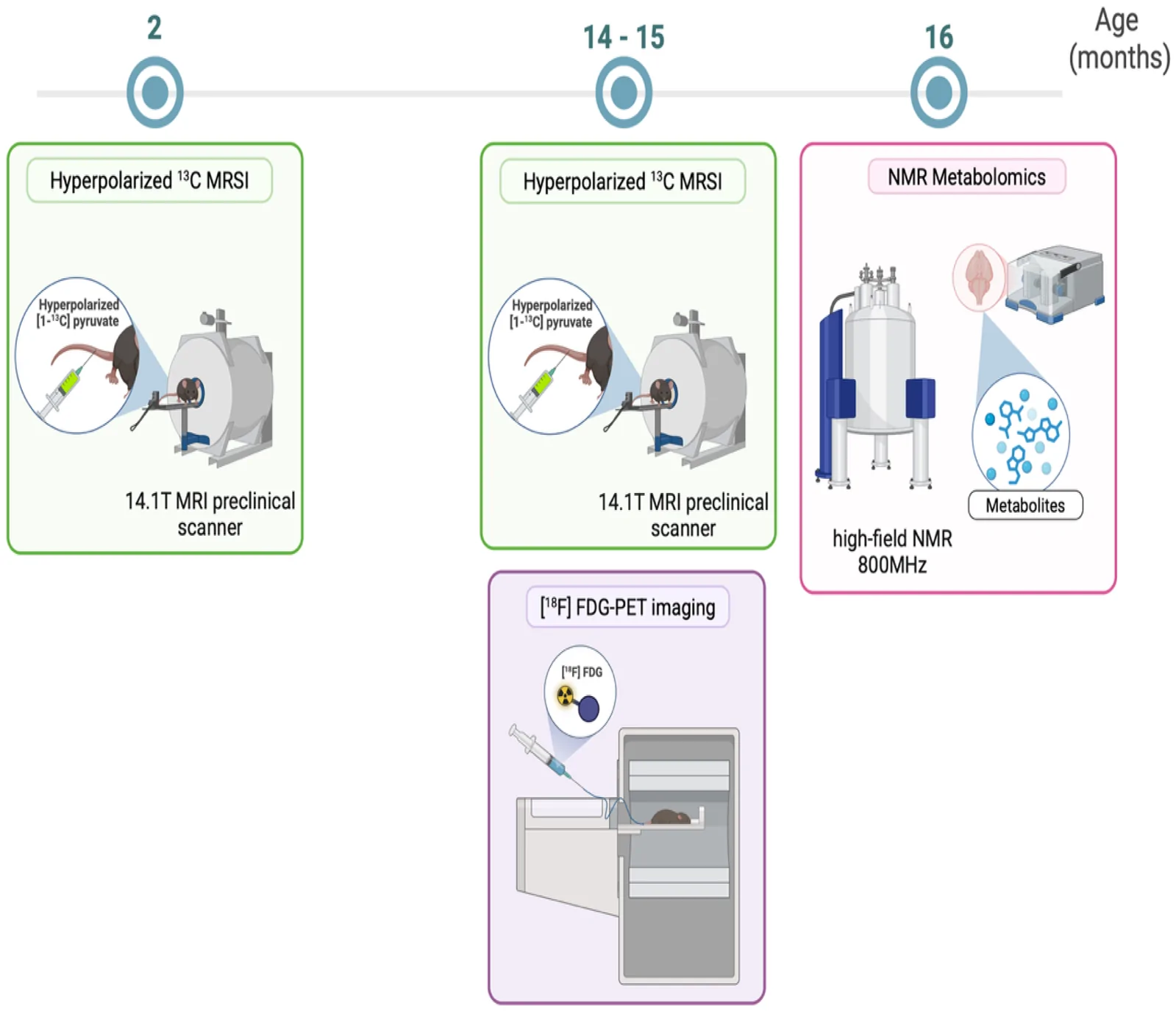
Experimental design using a multimodal approach to assess metabolic alterations in the hAPP-J20 mouse model. Created in https://BioRender.com.

In this study, we used the hAPP-J20 mouse model that presents an overexpression of the Swedish (K670N and M671L) and Indiana (V7171F) mutations of the human amyloid precursor protein (hAPP)^1,2^. Age-matched hAPP-J20 mice were housed with littermate wild-type (WT) controls, including both females (WT n = 11; hAPP-J20 n = 13) and males (WT n = 11; hAPP-J20 n = 13), in a 12 hour dark/light cycle in controlled temperature, supplied with food and water ad-libitum (Fig. 1A). HP ^13^C MRSI was performed longitudinally at both 2 and 14 months of age, [^18^F]FDG-PET at 14–16 months and *ex vivo* NMR metabolomics at the same age, following the last imaging session.

### HP C MRSI Imaging

#### Preparation of HP ^13^C Pyruvate and Urea

24μl [1–^13^C]pyruvate sample (pyruvic acid, 15mM OX63 tritylradical [Oxford Instruments] and 1.5mM Gad-DOTA) and 55μl ^13^C urea (6.4 M urea in glycerol, with 23mM OX63 trityl radical) were co-polarized for ~ 1 hour in a Hypersense polarizer (Oxford Instruments, 3.35 Tesla) and rapidly dissolved in a 4.5 ml heated buffer (80mM NaOH in Tris with EDTA) to give a pH 7 solution of 80mM pyruvate and 78mM urea. Immediately following dissolution, 300μl of this HP solution was injected via the tail vein catheter over 12 seconds.

#### Data acquisition

All *in vivo* MR experiments were conducted on a 14.1 Tesla vertical MR system (Agilent Technologies, PaloAlto, CA) equipped with 100 G/cm gradients and a dual tune ^1^H-^13^C volume coil (ØI = 40 mm). For each imaging session, mice were anesthetized using isoflurane (2% in O_2_) and a 27 G catheter was secured in the tail vein to allow for intravenous (iv) injection of the HP probes. Animals were then positioned in a dedicated cradle under constant anesthesia and placed in the MR bore; respiration was continuously monitored during all acquisitions to ensure animal well-being. First, axial T2-weighted images were acquired for adequate positioning of the grid used for HP ^13^C acquisitions. The following parameters were used: echo time (TE)/ repetition time (TR) = 20/1200 ms, slice thickness = 1.8 mm, number of averages = 2, matrix = 256 × 256, field of view (FOV) = 30 × 30 mm^2^. For HP ^13^C MRSI acquisitions, [1-^13^C]pyruvate and ^13^C Urea preparation (as described above) was hyperpolarized using a Hypersense DNP polarizer (Oxford Instruments) for approximately one hour. 2D ^13^C MRSI data were acquired 18 sec after iv injection of the HP [1-^13^C]pyruvate and ^13^C Urea solution, using the following parameters: TE/TR = 0.56/68 ms; spectral width = 4006 Hz; 256points; matrix 16x16, in-plane resolution = 1.9 × 1.9 mm^2^, flip angle (FA) = 10°; FOV = 30 × 30 mm^2^; 5 mm slice thickness.

#### Data analysis

HP ^13^C MRSI datasets were analyzed using the SIVIC software (http://sourceforge.net/apps/trac/sivic/) and custom-built programs written in MATLAB (MATLABR2011b, The MathWorks Inc.). The k-space dimensions were zero-filled by a factor of two resulting in a 32 × 32 matrix. The area under the curve (AUC) of HP [1-^13^C]pyruvate, HP [1-^13^C]lactate, and HP ^13^C urea Lorentzian fits were measured, and the corresponding metabolite ratio (HP [1-^13^C]lactate/pyruvate) was calculated for each voxel. Regions of interest (ROIs) including the cortex, hippocampus and subcortical area were defined using the T2-weighted images and HP data were reported as the mean SNR of the corresponding voxels (Fig.S1). Blood voxel SNR was calculated from the extracranial vessel as the ^13^C Urea peak SNR from the voxel containing the highest intravascular ^13^C Urea signal divided by the standard deviation of noise measured from a signal-free spectral region. To account for individual variability in substrate delivery, metabolite signals were subsequently normalized to the vascular ^13^C Urea voxel to correct for individual differences in substrate delivery, thereby reflecting the apparent metabolic change relative to vascular substrate availability.

### [^18^F]FDG PET imaging

#### Data acquisition

All scans were performed on a dedicated small animal PET/computerized tomography (CT) scanner (Inveon, Siemens Healthcare, Malvern, PA, USA). Mice received 71 ± 4.5μCi of [^18^F]-FDG in 0.1 mL volume iv *via* tail vein. Fifty-five minutes after radiotracer administration, static PET images were collected (10-minute acquisition time), followed by a 10-min CT acquisition for attenuation correction of PET reconstruction. Animals were kept awake at room temperature during the 55 minutes uptake time. The animals were placed under anesthesia (2% isoflurane in O_2_) during the injections and scans.

### Data analysis

PET images were reconstructed using the ordered subsets expectation maximization (OSEM) algorithm, provided by the manufacturer. The resulting PET images had a 128 x 128 x 159 matrix with a voxel size of 0.776 x 0.776 x 0.796 mm^3^. CT images were reconstructed using a cone-beam Feldkamp reconstruction algorithm (COBRA, Exxim Computing Corporation, Pleasanton, CA, USA). The reconstructed CT images had a 512 x 512 x 662 matrix size with an isotropic voxel size of 0.191 mm^3^.The co-registered attenuation map from CT, obtained via a pre-derived rigid transformation matrix, was used for attenuation correction of the PET data. A total of 15 ROIs were automatically delineated using VivoQuant software and corresponding mean percent-injected dose per grams(%ID/g) values were obtained. Static [^18^F]FDG-PET images were quantified using the standardized uptake value (SUV) to provide a semi-quantitative measure of regional glucose uptake.

#### Ex vivo NMR metabolomics

### Tissue processing

A portion of the animals at the last time point were allocated for *ex vivo* NMR metabolomics. Brains from both females (WT n = 5, hAPP-J20 n = 4) and males (WT n = 5, hAPP-J20 n = 4) were snap frozen after euthanasia. For perfusion, animals were given an overdose of ketamine and xylazine before transcardial perfusion with ice-cold saline. The brain was then removed, and each hemisphere was separated and snap frozen. All tissue was stored at −80°C prior to future *ex vivo* analysis. Frozen tissue samples were maintained on dry ice throughout processing to preserve metabolic integrity, and all solvents (methanol and chloroform) were pre-cooled prior to use. Tissue was homogenized in cold methanol using bead-based mechanical disruption, followed by a biphasic extraction with chloroform and water, vortexing, and centrifugation to separate phases; the aqueous (polar metabolite) fraction was collected, snap-frozen, and lyophilized for 48 h. The final NMR extract concentration was 600μl including TSP in D_2_0 as a chemical reference.

### Data acquisition

Data were acquired with a Bruker Avance NEO 800 MHz spectrometer using an actively shielded Z-gradient 5-mm TCI cryoprobe at 25°C. Spectra were acquired using programs from the TopSpin v.4.0.8 pulse program library (TopSpin v.4.0.6), specifically, the noesygppr1d sequence with the following parameters: FID 64k points (TD) RG auto-adjusted, recycle delay (D1) of 4s, NOESY mixing time of 10ms (D8), 4 dummy scans, 352 scans (NS), with a total duration of 32min 9s. Other parameters such as the power of presaturation and the amplitude of crusher gradients are set according to the specification of the pulse sequence. In addition, a subset of the samples was used to acquire fully relaxed spectra with a long TR (~ 49 s; relaxation delay 48.6 s) to allow full T1 relaxation. These data were used for absolute concentration normalization for each metabolite.

### Data analysis

Preprocessing and analysis were performed in Chenomx 10.12 (Chenomx Inc., Edmonton, AB, Canada). Preprocessing steps included phase and baseline correction and chemical shift calibration. A total of 36 metabolites were fitted – each integral fit for each selected peak cluster was visually inspected and corrected. To ensure consistency, the same selected peaks were fitted across all samples for each metabolite (Fig. S2). Each metabolite concentration was normalized to the sample’s weight and using the average concentrations of the metabolite’s full T1 relaxation. These data were further used for: orthogonal Partial Least Squares Discriminant Analysis (OPLS-DA), and Pathway Enrichment analysis, all performed in MetaboAnalyst 6.0(28). Moreover, a correlation-based metabolic network analysis was performed to assess metabolic (rewiring), done in Matlab 2024a (Mathworks, Natick, MA).

### Statistical analysis

Statistical analysis for HP ^13^C MRSI and [^18^F]FDG PET were performed in GraphPad Prism 10.6 (GraphPad Software, LLC). Data were analyzed for each sex separately. For HP ^13^C MRSI, a linear mixed model was applied, using genotype and age as main effects and the genotype X age interaction (*p* < 0.05). If a significant interaction was present, post-hoc testing was applied using post-hoc Šidák correction. When no interaction was observed, it was removed from the model and further tested for significance of main effects (age or genotype only). For the [^18^F]FDG PET imaging data, multiple Mann-Whitney tests were applied for all regions with FDR correction (*p* < 0.05), for each sex separately. For the NMR metabolomics network analysis, within group significant metabolic pairs were calculated based on a Pearson t-test, testing for correlations that are ≠ 0. Inter-genotype differences were assessed using a Fisher z-transform of Pearson correlations and comparing Z-transformed correlations using a standard normal approximation for only significant pairs in WT and/or hAPP-J20 groups, per sex. All comparisons were corrected for multiple testing, using Benjamini-Hochberg FDR (*p < 0.05*).

## Results

**Figure 2:**
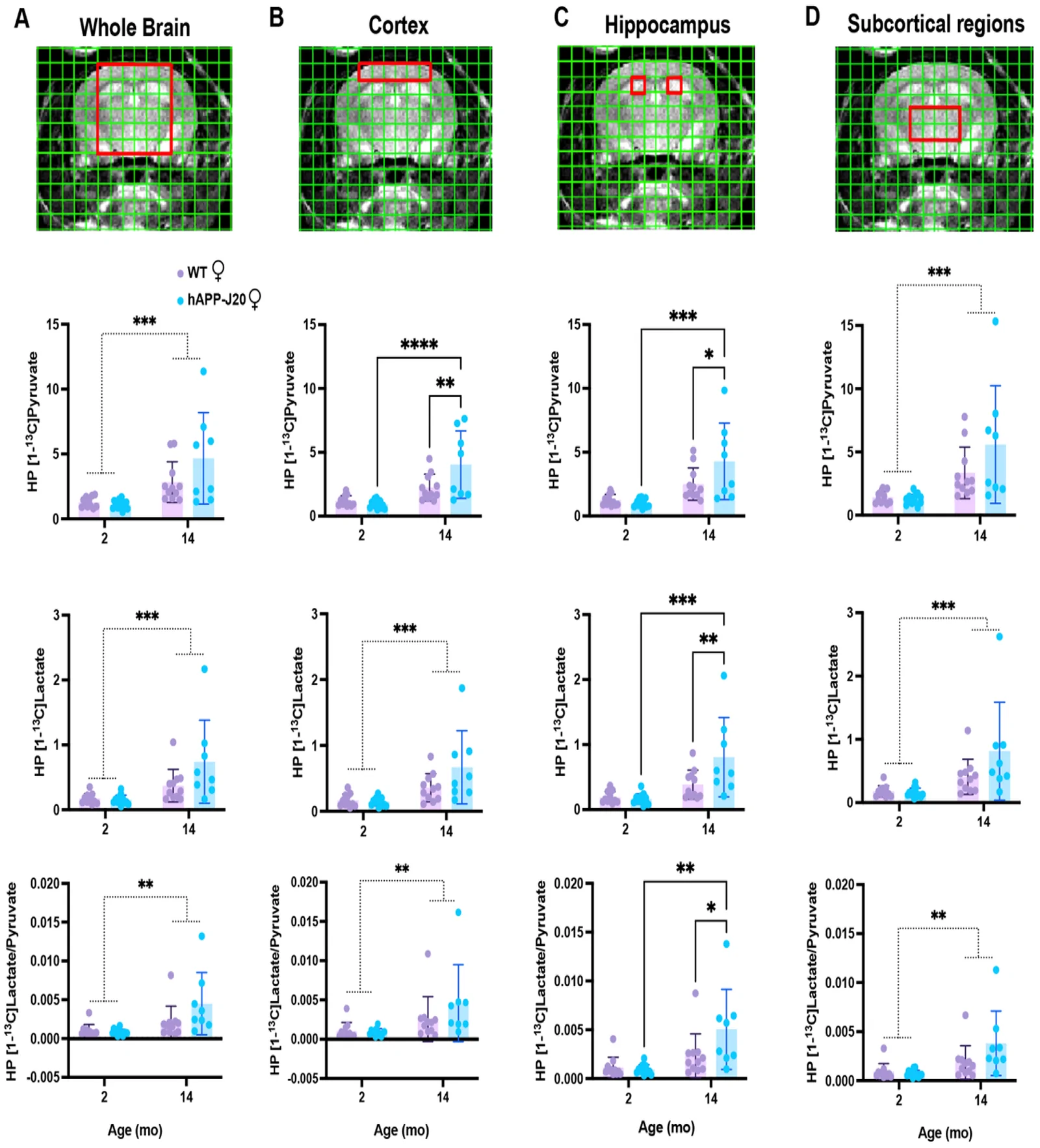
HP ^13^C MRSI reveals hippocampal alterations in hAPP-J20 females. Each column represents the anatomical image with an overlay ROI, whereas inter-genotypic differences in normalized HP [1-13C]Pyruvate, HP [1-13C]Lactate and HP [1-13C]Lactate/Pyruvate conversion, for (A) whole brain, (B) Cortex, (C) Hippocampus, and (D) Subcortical regions. Graph representation of bar graphs with subject values with SD per group; linear mixed model with Šidák post-hoc correction: dotted line - main age effect, full line - post-hoc comparisons; *p ≤ .05, **p ≤ 0.01, ***p ≤ 0.001, ****p ≤ 0.0001

**Figure 3:**
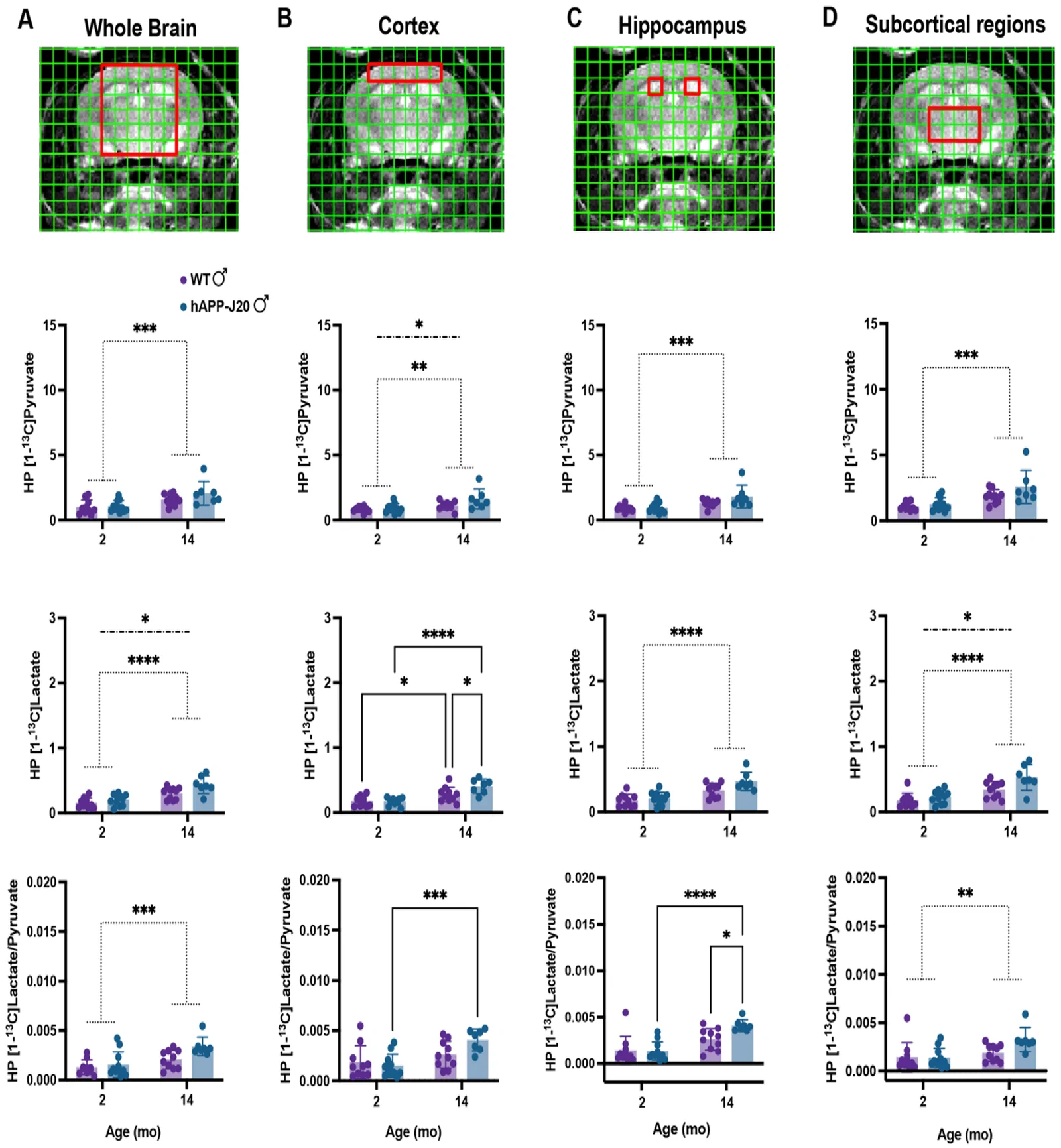
Cortical progressive metabolic alterations in hAPP-J20 male mice. Each column represents the anatomical image with an overlay ROI, whereas inter-genotypic differences in normalized HP [1-13C]Pyruvate, HP [1-13C]Lactate and HP [1-13C]Lactate/Pyruvate conversion, for (A) whole brain, (B) Cortex, (C) Hippocampus, and (D) Subcortical regions. Graph representation of bar graphs with subject values with SD per group; linear mixed model with Šidák post-hoc correction: dotted line - main age effect, full line - post-hoc comparisons; *p ≤ .05, **p ≤ 0.01, ***p ≤ 0.001, ****p ≤ 0.0001

To assess age- and genotype-dependent changes in brain metabolism, region-specific (cortex, hippocampus and subcortical regions) HP ^13^C Pyruvate MRSI was performed in male and female WT and hAPP-J20 mice at 2 and 14 months of age. Representative grid overlays illustrate the spatial coverage used for quantification over the whole brain and specific brain regions (Fig. 2, Fig. 3).

### HP ^13^C MRSI identifies a hippocampal glycolytic phenotype in female hAPP-J20 mice

To assess whether genotype effects were driven by differences in substrate delivery, we first examined the unadjusted HP [1-^13^C]Pyruvate and [1-^13^C]Lactate SNR (Fig. S3). No genotype-dependent differences were observed in whole brain or across regions, indicating comparable HP signal delivery and detection between groups. Consistent with this, perfusion assessed using HP ^13^C urea showed no significant differences, further supporting the absence of delivery-related effects (Fig. S5). Following the blood delivery normalization, in the whole brain, a significant aging effect (*p* < 0.001) was observed in both apparent [1-^13^C]Pyruvate and lactate as well as their metabolic flux, HP [1-^13^C]Lactate/Pyruvate, showing a progressive increase with age, irrespective of genotype (Fig. 2A). When assessing region-specific alterations, the aging effect was also present, however, in the hippocampus, a significant increase in both apparent [1-^13^C]Pyruvate and lactate metabolites and HP [1-^13^C]Lactate/Pyruvate - the flux proxy for glycolysis, was observed in the hAPP-J20 group both with age (*p* < 0.05 – 0.01) and as compared to WT at 14 months of age (*p* < 0.05; Fig. 2C).

### HP ^13^C MRSI reveals a cortical shift towards glycolytic metabolism in male hAPP-J20 mice

In the males, when comparing the unadjusted metabolite signal, on the whole brain level, HP [1-^13^C]lactate SNR showed significantly higher levels in 2-month-old male hAPP-J20 mice compared to age-matched controls (*p* < 0.01; Fig. S4A). In addition, an age-dependent increase in HP [1-^13^C]lactate SNR was also seen only in the WT group (p < 0.01) from 2 to 14 months. Using ^13^C Urea, a proxy for perfusion, an overall aging effect with significant decrease was observed in the whole brain and more prominently in both the cortex and the hippocampus (*p* < 0.001), irrespective of genotype (Fig. S5B,C). When comparing the normalized metabolite signals, in the whole brain, the males showed a predominant aging effect with a progressive increase from 2 to 14 months of age in both metabolites (*p* < 0.001–0.0001), as well as the HP [1-^13^C]Lactate/Pyruvate flux (*p* < 0.001; Fig. 3A). Despite a lack of interaction, the apparent HP [1-^13^C]Lactate had a significant main genotype effect (*p* < 0.05) indicating overall differences between genotypes across ages. In the cortex, an overall change in the metabolites and their apparent glycolytic flux was observed, with a main genotype difference in HP [1-^13^C]Pyruvate (*p* < 0.05; Fig. 3B), and a significant increase from 2 to 14 months of age the hAPP-J20 group in both the normalized HP [1-^13^C]Lactate (*p* < 0.0001) and HP [1-^13^C]Lactate/Pyruvate flux (*p* < 0.001; Fig. 3B). In addition, a genotypic difference was present in the apparent HP [1-^13^C]Lactate at 14 months with higher lactate abundance in hAPP-J20 compared to WT (*p* < 0.05; Fig. 3B). Similar shift was observed in normalized glycolytic flux (HP [1-^13^C]Lactate/Pyruvate) in the hippocampus, showing an age-dependent increase (*p* < 0.0001) and genotype-specific increase in the hAPP-J20 as compared to the WT group (*p* < 0.05; Fig. 3C).

#### [ ^18^ F]FDG-PET reveals no differences in glucose uptake between hAPP-J20 mice and control animals, irrespective of the brain region

**Figure 4:**
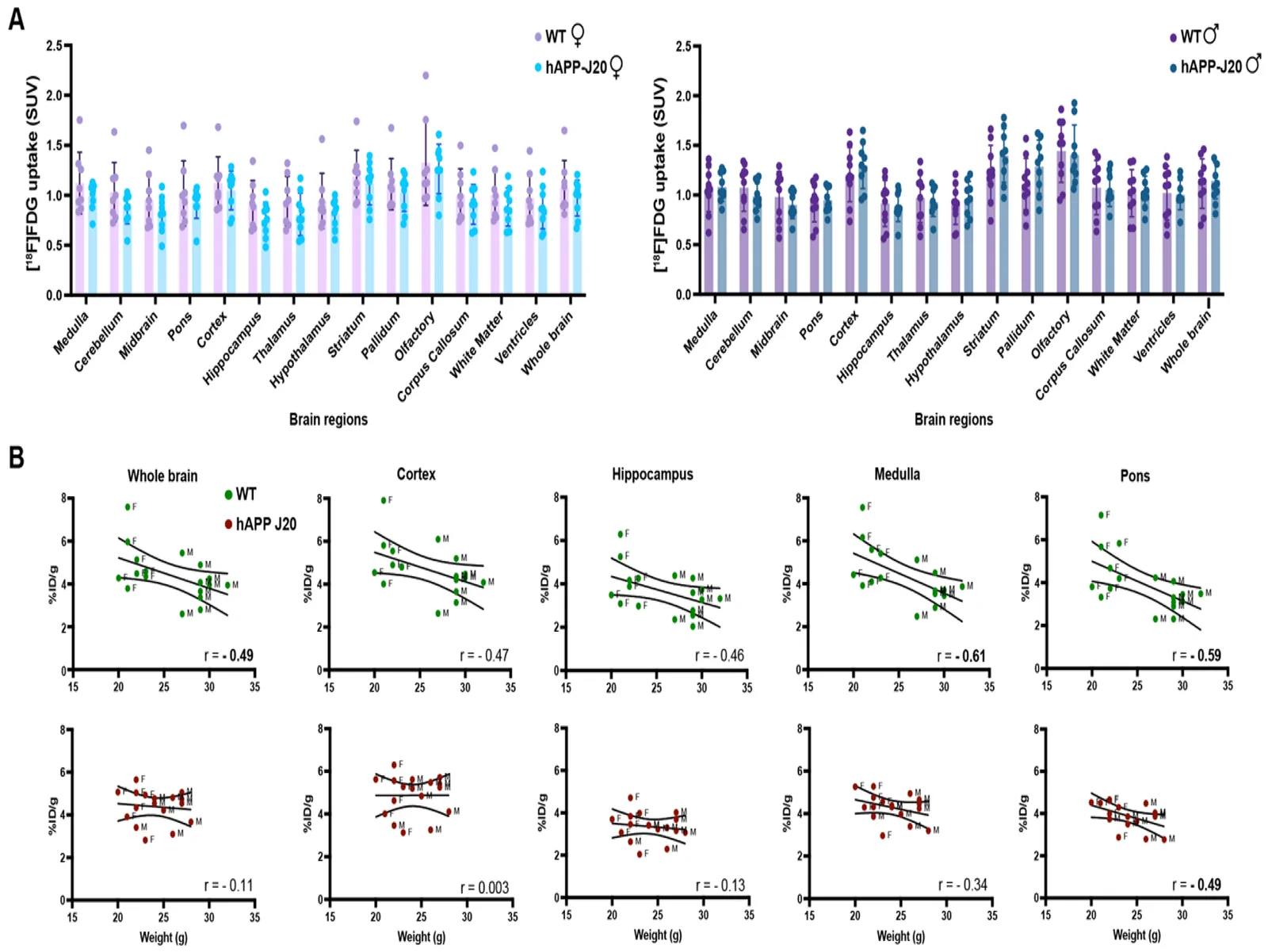
[^18^F]FDG PET brain glucose uptake is similar across between WT and hAPP-J20 brain regions, but loses sex- and weight-dependent scaling in hAPP-J20 mice (A) Comparisons of SUV across multiple brain regions between WT and hAPP-J20 mice in each sex; Graph representation of subject values with SD per group, multiple Mann-Whitney tests (FDR corrected, *p* < 0.05); (B) Correlation plots between %ID/g and body weight across different brain regions, shown for each genotype; F – female, M – male.

To determine how conventional metabolic techniques such as [^18^F]FDG PET compared to HP ^13^C MRSI, we next measured glucose uptake at the last imaging time point (15 months), in both WT and hAPP-J20 females and males, across different brain regions. Comparisons of SUV across multiple regions in both sexes showed no significant difference in glucose uptake (Fig. 4A). Because SUV normalizes uptake to body weight, we next examined the underlying relationship between body weight and non-normalized brain [^18^F]FDG uptake (%ID/g). In the WT group, a significant negative correlation between body weight and FDG uptake was observed in the whole brain, specifically pronounced in medulla and pons as well as a trend in cortex and hippocampus (Fig. 4B). This correlation also revealed a clear separation between females and males. In contrast, in the hAPP-J20 mice, there was no significant correlation between weight and [^18^F]FDG uptake in the whole brain, and the clear separation between female and male, as seen in the WT group, was largely lost (Fig. 4B).

### OPLS-DA analysis of NMR untargeted metabolomics reveals sex-specific metabolic rewiring in hAPP-J20 mice

**Figure 5:**
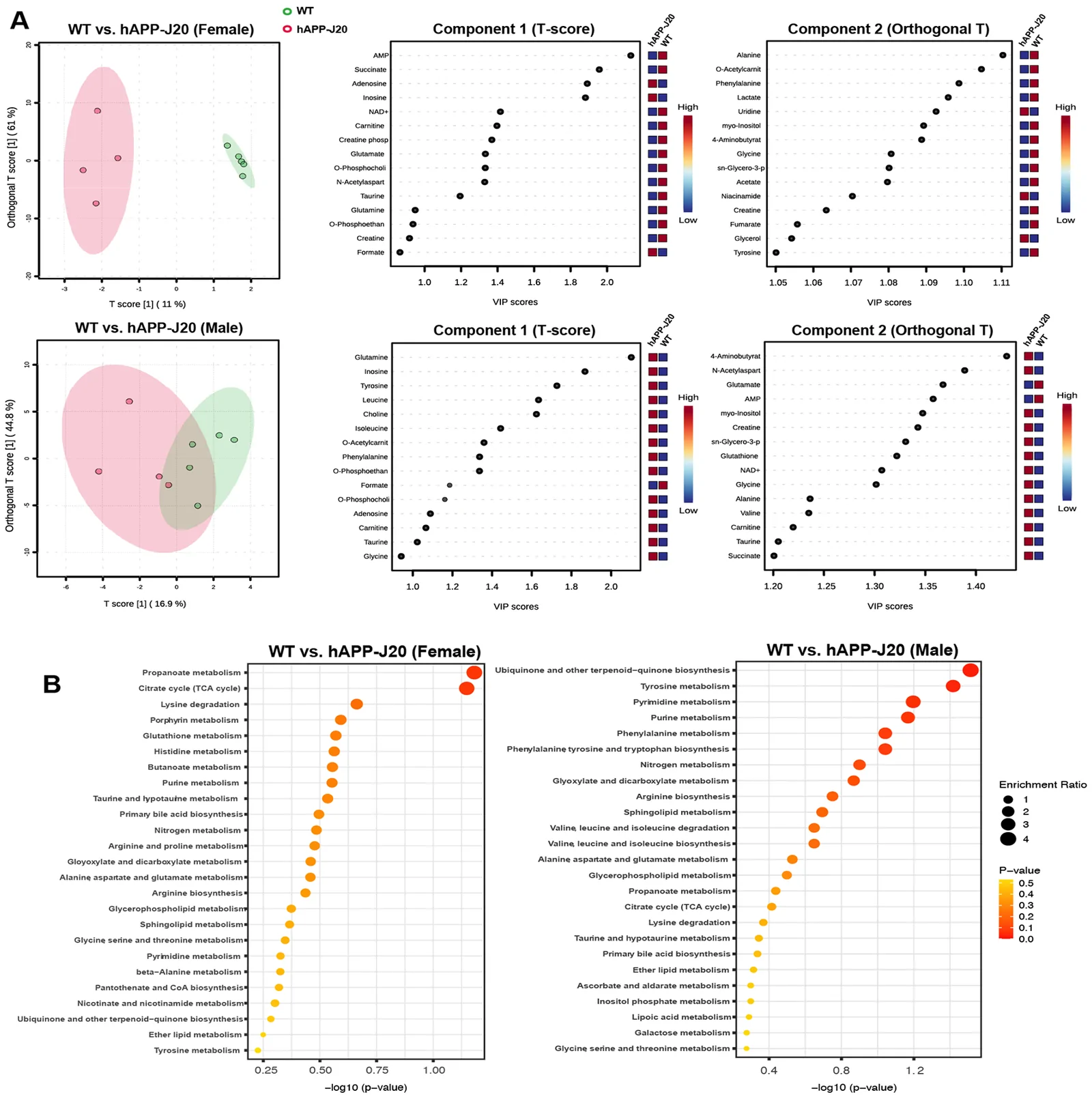
Metabolomics landscape reveals sex-specific metabolic impairments in hAPP-J20 mice (A) OPLS-DA T-score for WT vs. hAPP-J20 for each sex and the % of explained variance that is able to successfully discriminate WT from hAPP-J20 females, but not males; (B) Enrichment analysis based on the KEGG Database for top 25 metabolic pathways based on most affected metabolic processes between genotypes within each sex.

The concentrations of the 36 metabolites quantified in the whole brain extracts are reported in the supplementary information tables. In females, OPLS-DA analysis showed a clear distinction between WT and hAPP-J20 (predictive T-score 11%), especially showing tight clustering within the WT group, indicative of a homogeneous metabolic profile (Fig. 5A). The VIP analysis indicated that the group discrimination is primarily driven by metabolites linked to purine metabolism (ATP, Adenosine, Inosine) as well as TCA cycle metabolites, such as succinate. In contrast, the orthogonal component was largely driven by the intra-group heterogeneity in the hAPP-J20 group (predictive T-score 61%), suggesting greater metabolic variability in the hAPP-J20 females. Metabolites contributing to this orthogonal variation included: glycolytic (alanine, lactate), and mitochondrial/fatty acid (O-acetylcarnitine) metabolites and the glial osmolyte, myo-Inositol, indicating individually divergent signatures of energy redistribution.

In males, OPLS-DA analysis shows greater overlap between WT and hAPP-J20, with neither of the groups forming a compact cluster (predictive T-score 16.9%; Fig. 5A). The VIP score of the predictive component reveals that, the genotype differentiation is largely due to amino acid (glutamine, tyrosine, leucine), and purine metabolism, whereas the orthogonal component (predictive T-score 44.8%) is driven by neurotransmitter-related (4-Aminobutyrate, Glutamate, N-Acetylaspartate) and purine metabolism (AMP). Notably, this orthogonal variance was not genotype-specific, suggesting that males exhibit higher baseline metabolic diversity independent of hAPP-J20 pathology.

### Pathway enrichment analysis shows sex-specific altered metabolic pathways in hAPP-J20 mice

In females, the two main pathways that were significantly altered in hAPP-J20 mice compared to WT were propanoate metabolism (1 hit: Succinate) and the TCA cycle (2 hits: Succinate and Fumarate; Fig. 5B). These results indicate a targeted alteration in the mitochondrial carbon flux in hAPP-J20 female mice. In contrast, in males, the two main pathways that were significantly altered in hAPP-J20 mice compared to WT were the Ubiquinone and other terpenoid-quinone biosynthesis (1 hit: Tyrosine) and Tyrosine metabolism (2 hits: Tyrosine and Fumarate; Fig. 5B).

### Correlation-based metabolic network analysis shows genotype- and sex-distinct energetic integration and rewiring in hAPP-J20 mice

**Figure 6:**
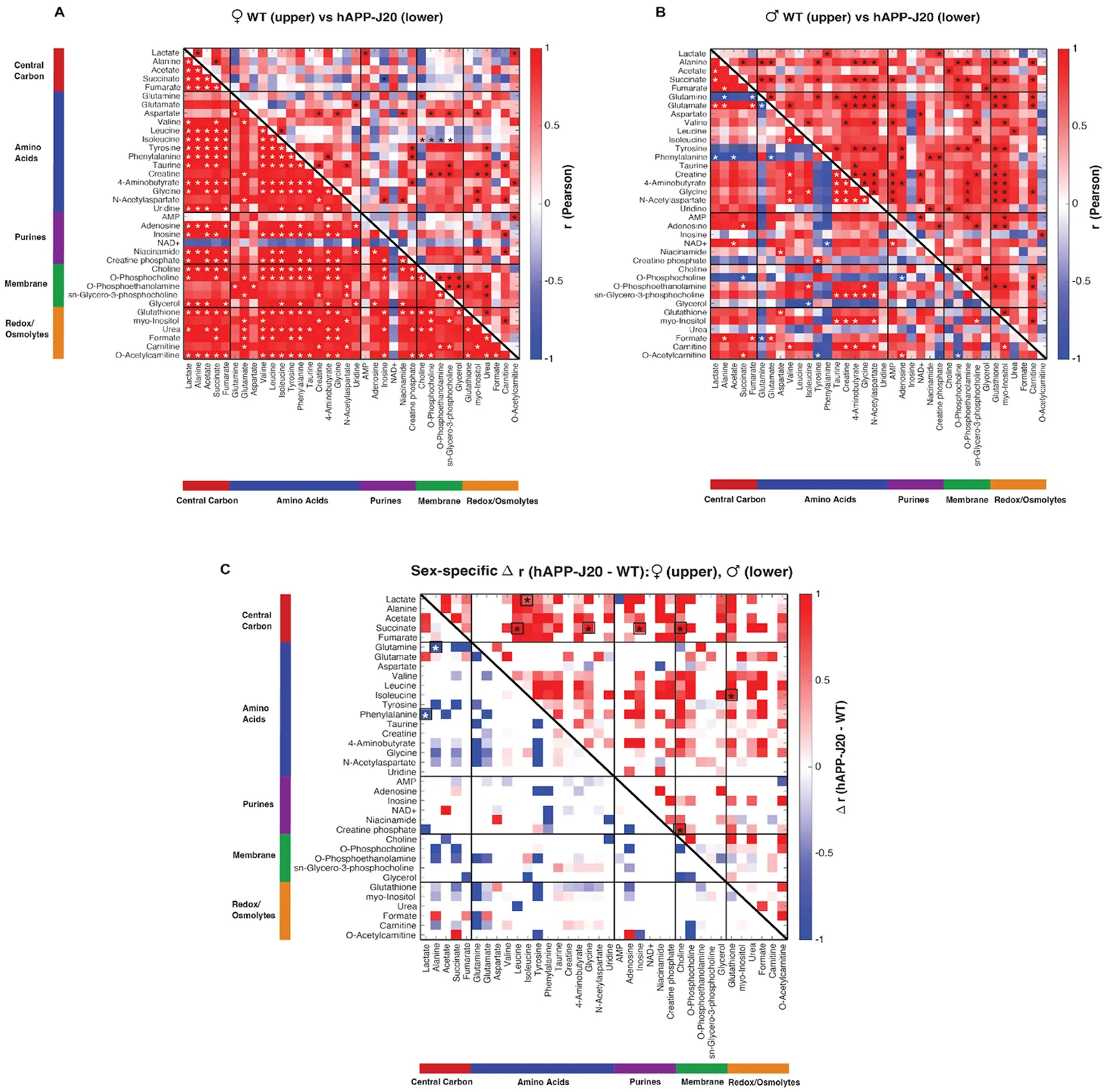
Metabolite-metabolite interactions in different KEGG subnetworks represent genotype- and sex-distinct energetic integration and rewiring in hAPP-J20 mice. (A,B) Pearson (r) correlation matrices in males and females, showing within group metabolic connectivity in WT (upper triangle) and hAPP-J20 (lower triangle) and significant metabolic pairs (t-test with r ≠ 0, p < 0.05, uncorrected); (C) Genotype differences in metabolic edges within each sex, measured for Fisher z-transformed correlations, based on significant metabolic pairs in WT and/or hAPP-J20 group, per sex (upper triangle: females, lower triangle: males), with multiple comparisons correction (p < 0.05, FDR corrected); Metabolite order in matrices is based on KEGG Pathway Assignments.

In females, the WT group displayed mostly pathway-restriction (amino acids – membrane – osmolytes), with significant association occurring in smaller clusters rather than uniformly across the full matrix (Fig. 6A). In contrast, the hAPP-J20 female group exhibited a globally strengthened correlation matrix (*p* < 0.05, uncorrected), characterized by dense and positively significant correlations across nearly all metabolic classes (Fig. 6A). In males, this correlation pattern was reversed, where metabolites within the WT group displayed stronger correlations, whereas the hAPP-J20 males exhibited weaker and more diverse interactions (p < 0.05, uncorrected; Fig. 6B). To quantify genotype-specific rewiring, we assessed the correlation differences between hAPP-J20 and WT groups per sex. Female hAPP-J20 mice showed significantly stronger metabolic edges as compared to WT, which were mostly centered around Succinate, a potential central hub of metabolic rewiring (Fisher z-test, p < 0.05, FDR corrected; Fig. 6C). In males, only 2 metabolic edges show significant changes in the hAPP-J20 group with reduced correlation, as compared to WT, that indicate disruption in the glycolysis – amino acid metabolism cycling (Fig. 6C).

## Discussion

In this study, we combined HP ^13^C MRSI, [^18^F]FDG-PET, and ex vivo ^1^H-NMR metabolomics to characterize how Aβ pathology reshapes brain energy metabolism in the hAPP-J20 model. First, HP ^13^C MRSI revealed a progressive, region-specific glycolytic flux in hAPP-J20 mice, manifesting as an increased apparent HP [1-^13^C]Lactate/Pyruvate conversion that localized to the hippocampus in females and to the cortex and hippocampus in males. Second, this glycolytic signature was detected by HP ^13^C MRSI at a stage when static [^18^F]FDG-PET showed no genotype difference in regional glucose uptake, although the normal sex- and weight-dependent structure of FDG uptake was lost in hAPP-J20 mice. Third, ex vivo metabolomics uncovered sex-divergent metabolic rewiring, centered on succinate and the TCA cycle in females and on tyrosine/redox-linked pathways in males. Together, these data position HP ^13^C MRSI as a sensitive, complementary readout of metabolic dysfunction in Aβ pathology that captures alterations in metabolic flux not visible to glucose-uptake imaging alone.

The progressive increase in conversion in the HP [1-^13^C]Lactate/Pyruvate metabolic flux in the hAPP-J20 mice is consistent with a growing literature linking aerobic glycolysis to Aβ-vulnerable brain regions and Aβ deposition(14, 29). In preclinical models, converging evidence suggests that Aβ pathology is accompanied by lactate-associated metabolic remodeling, including altered astrocyte–neuron lactate shuttle activity and increased glycolysis-derived lactate production(30-32). Cellular and human-tissue studies indicate that Aβ actively drives this metabolic phenotype: exposure of astrocytes to Aβ increases glucose uptake, GLUT1 membrane localization, glycolytic rate, and lactate release, while compromising mitochondrial integrity(33, 34). Our in vivo observation of elevated HP [1-^13^C]Lactate labeling in hAPP-J20 mice is the flux-level correlate of these findings and aligns with the elevated hippocampal interstitial lactate reported in the APP/PS1 amyloidosis model(30, 35). Importantly, because the unadjusted HP ^13^C signal, ^13^C Urea-based perfusion, and substrate delivery did not differ between genotypes, the increased apparent conversion is most parsimoniously attributed to altered enzymatic flux through lactate dehydrogenase (LDH)(16, 18, 36-38) rather than to differences in pyruvate delivery(38).

The regional localization of the glycolytic phenotype is biologically informative. In females the effect was strongest in the hippocampus, the structure earliest and most severely affected in the hAPP-J20 model, where CA1 neuronal loss, microglial activation, and gliosis emerge before overt plaque deposition(26). The hAPP-J20 brain is also characterized by aberrant network hyperexcitability and spontaneous epileptiform activity originating in hippocampal and cortical circuits(25, 27), and chronic neuronal activation upregulates glycolytic flux and lactate production to meet activity-dependent energy demand(21).

An activity- and inflammation-driven increase in glycolysis therefore provides a coherent mechanism linking the known circuit pathology of this model to the HP ^13^C signature we observe. That the dominant region differs by sex—hippocampal in females, cortical in males—further suggests that Aβ engages partially distinct regional and cellular programs in the two sexes, a theme reinforced by the metabolomic data.

A central message of this work is the complementarity between HP ^13^C MRSI and [^18^F]FDG-PET. FDG-PET, the clinical gold standard for metabolic imaging in AD, reports glucose transport and phosphorylation but is blind to the downstream partitioning of pyruvate between glycolytic and oxidative fates(13, 14). In our study, regional FDG uptake did not differ between genotypes, yet HP ^13^C MRSI detected a clear glycolytic flux increase—indicating that the abnormality lies not in how much glucose enters the tissue but in how pyruvate is subsequently metabolized. This dissociation echoes observations in the APP/PS1 model, where bulk lactate measures appeared stable while interstitial lactate and glycolytic enzyme expression were altered(30, 35), and it underscores that flux-based and uptake-based readouts capture different facets of metabolic dysfunction. The notable loss of the normal sex- and weight-dependent relationship of FDG uptake in hAPP-J20 mice, despite preserved mean SUV, is consistent with this interpretation: Aβ pathology perturbs the regulation of brain glucose handling in ways that mean SUV alone can miss, reinforcing the value of adding a dynamic, flux-sensitive probe. The absence of a genotype effect on FDG uptake in hAPP-J20 mice stands in contrast to the global reductions in CMRglc reported in the majority of rodent amyloidosis models, as reviewed by Ni et al. (2021), and may reflect the known sensitivity of [^1^⁸F]FDG to experimental conditions including anesthesia, age, and sex, as well as the potential confounding influence of neuroinflammation-driven microglial glucose uptake, which may mask or counteract neuronal metabolic deficits at the level of mean regional SUV(39).

Interpreting the directionality of brain lactate changes requires careful consideration of context, as elevated and reduced lactate signals have both been reported in neurological disease and aging, reflecting distinct underlying pathophysiological states. In amnestic mild cognitive impairment, proton MRS studies report elevated posterior cingulate lactate that scales with cognitive impairment and hippocampal atrophy and is interpreted as a compensatory response to mitochondrial dysfunction(40). Conversely, HP ^13^C studies of normal human aging report a significant decline in normalized [1-^13^C]Lactate and [^13^C]Bicarbonate production labeling with age(41), as well as a study reporting a decline in aerobic glycolysis across the lifespan in healthy individuals(14). In this study, metabolite signals were normalized to a vascular HP ^13^C urea signal, such that our normalized values reflect apparent metabolite uptake and metabolism relative to vascular substrate delivery. Notably, HP ^13^C urea signal showed a significant age-related decline in both WT and hAPP-J20 mice equally, with no genotype-specific differences. This has two important interpretive consequences. First, the age-related increases in normalized HP [1-^13^C]Pyruvate and HP [1-^13^C]Lactate signals observed in both genotypes likely reflect enhanced tissue extraction and metabolic utilization of pyruvate relative to a declining vascular supply — a measure that is complementary to, rather than contradictory of, the declining within-region conversion efficiency reported by Uthayakumar et al. in healthy humans, and may reflect a compensatory upregulation of tissue pyruvate extraction with aging. Second, and critically, because the urea vascular signal declines equivalently in both genotypes, the genotype-specific elevation of HP [1-^13^C]Lactate/Pyruvate ratio in hAPP-J20 mice — observed prominently in the hippocampus in females and the cortex in males — cannot be attributed to differences in vascular delivery or perfusion and instead reflects a genuine pathological upregulation of glycolytic flux that is superimposed on normal aging-related metabolic changes. The emerging picture is that Aβ pathology adds to, rather than follows, the normal aging trajectory of brain glycolysis, and that HP [1-^13^C]Lactate conversion may therefore serve as a disease-sensitive, rather than simply age-sensitive, metabolic biomarker.

**Figure 7:**
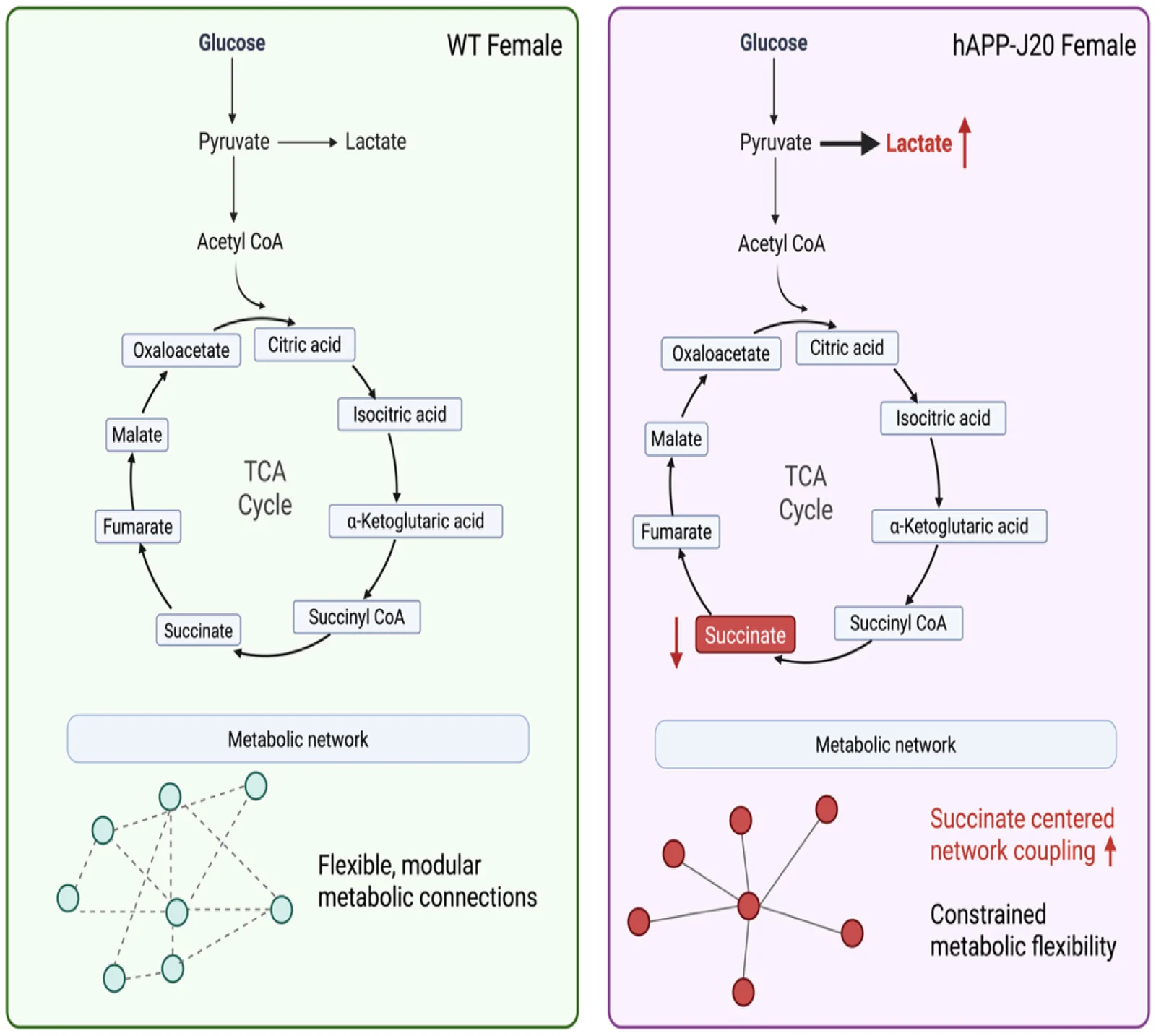
Schematic summary of glycolytic and metabolic-network remodeling in the female hAPP-J20 brain. In WT females (left), glucose-derived pyruvate feeds both lactate production and the TCA cycle, and the metabolic correlation network is flexible and modular, reflecting independently regulated metabolite pools. In hAPP-J20 females (right), apparent glycolytic conversion to lactate is increased while succinate is reduced, and the correlation network collapses into a rigid, succinate-centered configuration of strong, uniform coupling. Created in https://BioRender.com.

The ex vivo metabolomic analyses add mechanistic depth and reveal that the convergent glycolytic phenotype seen by imaging overlies sex-divergent steady-state remodeling. In females, OPLS-DA separated hAPP-J20 from the WT group, with the separation driven primarily by purine and energy metabolites—AMP, NAD^+^, adenosine, and inosine—alongside succinate, and pathway enrichment pointing to propanoate metabolism and the TCA cycle. The more robust change, however, was structural —succinate became the hub of a markedly strengthened correlation network, with relationships that were weak or anticorrelated in WT females collapsing into strong, uniform positive coupling. Because this coupling drew together metabolites moving in opposite directions—some lower (succinate, glycine), others higher (inosine)—it reflects a loss of independent metabolic regulation rather than co-suppression, consistent with the rigid, co-dependent central carbon metabolism increasingly described in the energetically compromised AD brain(42). Set against the hippocampal glycolytic shifts observed with the HP ^13^C imaging, this constrained oxidative profile points to a diversion of carbon away from the TCA cycle toward glycolysis in the Aβ-bearing female brain (Fig. 7).

In males, the signature was distinct and, in network terms, opposite. Rather than a strengthened, succinate-anchored network, hAPP-J20 males showed a loss of normal metabolic coupling, with strong positive correlations in WT—between lactate and phenylalanine, and between alanine and glutamine—reversing to strong negative correlations. At the abundance level, group discrimination centered on L-tyrosine, which was significantly elevated in hAPP-J20 males and links the two most enriched pathways, tyrosine metabolism and ubiquinone biosynthesis, rather than on a TCA-cycle signature. This combination of elevated tyrosine and decoupling of glycolytic and amino-acid nodes suggests a qualitatively different, less coordinated response than the network tightening seen in females. Such sex divergence is highly relevant to human AD, where women carry roughly two-thirds of disease burden and show sex-specific trajectories of glucose-metabolic decline and mitochondrial vulnerability(43, 44). The female-predominant hippocampal glycolytic phenotype and succinate-linked network remodeling reported here may therefore model, at the metabolic level, aspects of the heightened female susceptibility seen clinically, and argue for sex-stratified analysis in metabolic imaging studies of AD(45-47).

Finally, these results carry translational implications. HP [1-^13^C]pyruvate MRI is already in clinical use for oncological and neurological applications and, unlike PET, requires no ionizing radiation, enabling repeated longitudinal assessment(16, 18).Our demonstration that HP ^13^C MRSI detects an Aβ-associated glycolytic shift before FDG-PET registers a change supports its development as an early, flux-sensitive biomarker of metabolic dysfunction in AD, and as a pharmacodynamic readout for therapies aimed at brain energy rescue(5). Integrating dynamic flux imaging with uptake-based PET and steady-state metabolomics, as done here, offers a more complete account of metabolic pathophysiology than any single modality and provides a framework for tracking disease-modifying interventions.

### Limitations

Several limitations should be considered when interpreting these findings. First, we successfully quantified HP [1-^13^C]Pyruvate and HP [1-^13^C]Lactate, providing robust in vivo measures of cerebral glycolytic metabolism. HP [^13^C]Bicarbonate, the downstream product of pyruvate dehydrogenase (PDH) and a marker of mitochondrial oxidative metabolism, was not resolved in the present study. This likely reflects the challenges of performing HP ^13^C MRI at ultra-high field (14.1T), where the shorter T1 relaxation time of HP [1-^13^C]Pyruvate and its metabolites reduces the available signal lifetime for detecting lower-abundance products such as bicarbonate. Consequently, our measurements primarily characterize the glycolytic arm of pyruvate metabolism. Importantly, recent studies in humans at clinical field strengths have demonstrated reliable detection of brain [^13^C]Bicarbonate following HP [1-^13^C]Pyruvate administration, highlighting the feasibility of assessing oxidative metabolism in vivo and underscoring the translational potential of this approach(48, 49) Because brain [^13^C]Bicarbonate originates predominantly from PDH activity(50), incorporation of bicarbonate imaging in future clinical studies will provide a valuable opportunity to determine whether the glycolytic alterations observed here are accompanied by corresponding changes in mitochondrial oxidative metabolism. Second, we report the apparent HP [1-^13^C]Lactate/Pyruvate ratio - a widely used and straightforward metric that provides a robust measure of pyruvate-to-lactate labeling in vivo. While kinetic modeling can yield the apparent conversion rate constant (k_PL) and may offer a more quantitative assessment of label exchange by accounting for factors such as substrate delivery, acquisition timing, and relaxation effects(37), numerous preclinical studies have demonstrated strong agreement between lactate-to-pyruvate ratios and k_PL estimates across a range of biological conditions(51-54). In particular, work from Brindle and colleagues has shown that both metrics generally capture the same underlying metabolic changes and often lead to highly consistent biological interpretations(36, 37, 55). As our primary objective was to compare relative metabolic differences between groups under standardized acquisition conditions, the ratio-based approach provided a practical and biologically meaningful measure of HP ^13^C metabolic flux. Nevertheless, future studies incorporating dynamic acquisitions and kinetic modeling could further refine quantification and provide additional mechanistic insight into pyruvate metabolism. Finally, HP acquisitions were performed under isoflurane anesthesia. Although isoflurane can impact HP metabolite-to-substrate ratios in a dose-dependent manner(38); anesthesia was held constant across groups, ensuring reliable comparisons while maintaining standardized imaging conditions.

A further consideration is cellular specificity. HP ^13^C MRSI integrates signal across neurons, astrocytes, microglia and oligodendrocytes within each voxel and cannot attribute the observed lactate shift to a particular cell type(50) While our metabolomic data and the broader literature implicate a glial, inflammation-associated glycolytic program(35), cell-type-resolved approaches would be required to confirm the cellular source of the elevated lactate conversion.

Finally, this study leveraged the well-characterized hAPP-J20 model, which recapitulates several key features of Alzheimer's disease, including progressive Aβ pathology, neuroinflammation, and network hyperexcitability(25, 26), making it a valuable platform for investigating Aβ-associated metabolic alterations in vivo. The robust sex- and region-specific metabolic signatures identified here provide a strong foundation for future studies examining the generalizability of these findings across complementary models of neurodegeneration. Because the hAPP-J20 line does not develop tau pathology or substantial neurodegeneration and, as an overexpression model, may exhibit APP-fragment effects beyond those attributable to Aβ itself, extending these investigations to Aβ knock-in models, tauopathy models, and genetically diverse backgrounds will be particularly informative. Future studies incorporating genetic risk modifiers such as APOE4, which is known to influence both brain energy metabolism and microglial function(7) will further clarify the mechanisms underlying the metabolic phenotypes observed here and help determine the extent to which these signatures represent convergent or model-specific features of Alzheimer's disease pathophysiology.

## Conclusion

Together, these findings establish HP ^13^C MRSI as a flux-sensitive imaging approach capable of detecting sex- and region-specific glycolytic dysregulation in AD, revealing progressive genotype-dependent increases in pyruvate-to-lactate conversion that diverge from normal aging and align with known patterns of AD vulnerability. Given the clinical translation of hyperpolarized pyruvate imaging, this work supports HP ^1^³C MRI as a promising disease-sensitive biomarker for identifying early metabolic dysfunction and advancing metabolic staging in Alzheimer’s disease.

## Supplementary Material

This is a list of supplementary files associated with this preprint. Click to download.

- SupplTable1LMMStatisticsallHP13CMRSFDGPET.xlsx
- SupplTable2NMRmetabolomicsconcentrationsnormtosampleweight.xlsx
- SupplTable3NMRmetabolomicsCorrmatrixstatistics.xlsx
- SUPPLEMENTARYFIGURES.docx
